# Plasticity and mTOR: Towards Restoration of Impaired Synaptic Plasticity in mTOR-Related Neurogenetic Disorders

**DOI:** 10.1155/2012/486402

**Published:** 2012-04-30

**Authors:** Tanjala T. Gipson, Michael V. Johnston

**Affiliations:** ^1^Tuberous Sclerosis Center, Hugo Moser Research Institute, Kennedy Krieger Institute, Baltimore, MD 21205, USA; ^2^Department of Neurology and Developmental Medicine, Hugo Moser Research Institute, Kennedy Krieger Institute, Baltimore, MD 21205, USA; ^3^The Clinical Trials Unit, Hugo Moser Research Institute, Kennedy Krieger Institute, Baltimore, MD 21205, USA; ^4^Departments of Neurology, Johns Hopkins University School of Medicine, Baltimore, MD 21205, USA; ^5^Departments of Pediatrics, Johns Hopkins University School of Medicine, Baltimore, MD 21205, USA; ^6^Physical Medicine and Rehabilitation, Johns Hopkins University School of Medicine, Baltimore, MD 21205, USA

## Abstract

*Objective*. To review the recent literature on the clinical features, genetic mutations, neurobiology associated with dysregulation of mTOR (mammalian target of rapamycin), and clinical trials for tuberous sclerosis complex (TSC), neurofibromatosis-1 (NF1) and fragile X syndrome (FXS), and phosphatase and tensin homolog hamartoma syndromes (PTHS), which are neurogenetic disorders associated with abnormalities in synaptic plasticity and mTOR signaling. *Methods*. Pubmed and Clinicaltrials.gov were searched using specific search strategies. *Results/Conclusions*. Although traditionally thought of as irreversible disorders, significant scientific progress has been made in both humans and preclinical models to understand how pathologic features of these neurogenetic disorders can be reduced or reversed. This paper revealed significant similarities among the conditions. Not only do they share features of impaired synaptic plasticity and dysregulation of mTOR, but they also share clinical features—autism, intellectual disability, cutaneous lesions, and tumors. Although scientific advances towards discovery of effective treatment in some disorders have outpaced others, progress in understanding the signaling pathways that connect the entire group indicates that the lesser known disorders will become treatable as well.

## 1. Introduction

Brain plasticity, the developing brain's ability to change in response to either positive experiences or negative experiences, is a critical component of pediatric neurology. The major types of plasticity in the developing brain include *adaptive plasticity*—occurs in response to learning or recovering from injury or disability; *impaired plasticity—*results from brain injury due to an acquired or neurogenetic disorder; *maladaptive plasticity—*a plastic response leading to a new disorder; *plasticity as the brain's Achilles' heel—*a mechanism, such as selective vulnerability of neurons, which creates risk for injury [1]. Basic cellular mechanisms of plasticity include overproduction of neurons followed by reduction via apoptosis [2]; continued production of new cells from stem cells in the hippocampus and lateral ventricle throughout life [3]; activity-dependent synaptic plasticity through receptor trafficking; activity-dependent production of growth factors; overproduction of synapses and axodendritic connections followed by pruning, activity-dependent stabilization of dendrites and axons; regulation of DNA expression by epigenetic regulation [1]. Although there are many disorders associated with impaired plasticity, this paper will highlight the clinical features, neurobiology associated with dysregulation of mTOR, preclinical studies, and clinical trials in tuberous sclerosis complex (TSC), neurofibromatosis-1 (NF1), and fragile X syndrome (FXS), as well as phosphatase and tensin homolog hamartoma syndromes (PTHS), neurogenetic disorders linked by abnormalities in synaptic plasticity and mTOR (mammalian target of rapamycin) signaling.

## 2. Methods

Pubmed was searched using the following search strategies: mTOR and/or neurology; mTOR and/or plasticity; mTOR and/or TSC; mTOR and/or NF1; mTOR and/or FXS; mTOR and/or PTHS; plasticity and/or neurology; plasticity and/or TSC; plasticity and/or NF1; plasticity and/or FXS; plasticity and/or PTHS. Clinicaltrials.gov was searched by disorder without language or country of origin restrictions for active studies through 11/30/11.

### 2.1. Tuberous Sclerosis Complex (TSC)

#### 2.1.1. Clinical Features

Tuberous sclerosis complex (TSC) has an incidence of 1/6000 and may be defined clinically by the presence or absence of major and minor features associated with the disorder and genetically by spontaneous or inherited mutations in TSC1 or TSC2. Major neurologic features include brain lesions-subependymal nodules, subependymal giant cell astrocytomas, and cortical tubers, intractable epilepsy in 60–90% [4–6], autism in up to 61% [7, 8], intellectual disability in 45% [9], and self-injury in 10% [10]. TSC has also been associated with pulmonary, cardiac, and cutaneous lesions (Table 1).

#### 2.1.2. Neurobiology of mTOR Dysregulation

**Figure 1:**
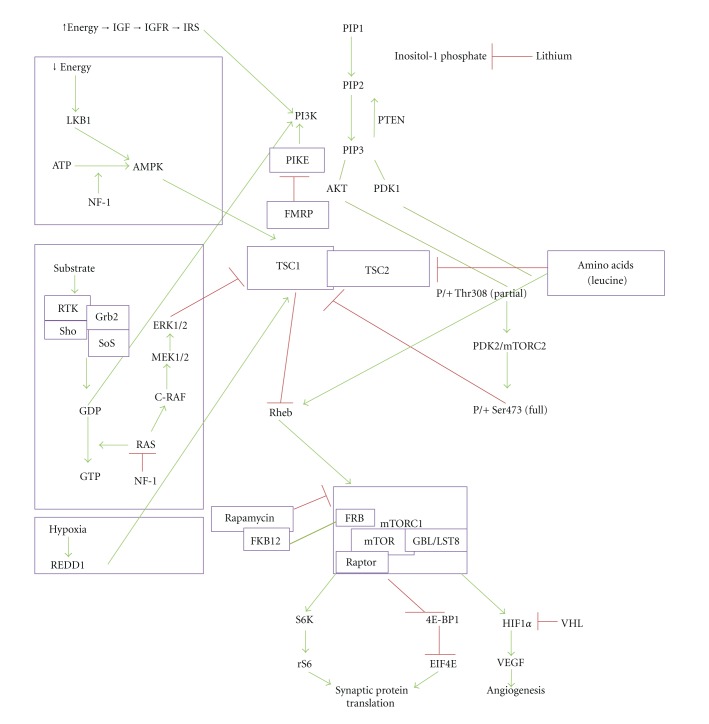
Pathways associated with neurogenetic conditions linked by mTOR and impaired synaptic plasticity. AKT-v—akt murine thymoma viral oncogene homolog 1; AMPK—adenosine monophosphate kinase; ATP—adenosine triphosphate; EIF4E/4EBP1—eukaryotic translation initiation factor 4E-binding protein 1; ERK—extracellular signal-related kinase; FKB12-FK506—binding protein family; FMRP—fragile X mental retardation protein; GBL/LST8—(mTOR-associated protein, LST8 homolog); GDP—guanosine diphosphate; GTP—guanosine triphosphate; GDP—guanosine diphosphate; HIF1*α*—hypoxia inducible factor 1, alpha subunit IGF—insulin-like growth factor; IGFR—insulin-like growth factor receptor; IRS—insulin receptor substrate; LKB1—serine threonine kinase 11; mTORC1—mammalian target of rapamycin complex 1; mTORC2—mammalian target of rapamycin complex 2; MEK—dual specificity mitogen-activated protein kinase 1; NF-1—neurofibromatosis 1; P/+ Thr308—phosphorylation of threonine, position 308; P/+ Ser473—phosphorylation of serine, position 473; PDK1—pyruvate dehydrogenase kinase, isozyme 1; PDK 2—pyruvate dehydrogenase kinase, isozyme 2; PI3K—phosphoinositide-3-kinase; PIKE—phosphoinositide 3-kinase enhancer; PIP1—p21-activated protein kinase-interacting protein 1 (PAK1 interacting protein 1); PIP2—phosphatidylinositol 4,5-biphosphate; PIP3—phosphatidylinositol (3,4,5)-triphosphate; PTEN—phosphatase and tensin homolog; RAS—Ras p21 protein activator 1 or RAS GTPase activating protein; REDD1—regulated in development and DNA damage responses; RHEB—Ras homologue expressed in brain; RTK—receptor tyrosine kinase; S6K—ribosomal protein S6 kinase; TSC—tuberous sclerosis complex; VEGF—vascular endothelial growth factor; VHL—von Hippel Lindau.

Overexpression of the serine/threonine protein kinase mammalian target of rapamycin (mTOR) results from disruption of either TSC1 or TSC2. Typically, TSC1 and TSC2 form a complex, which inhibits Rheb (ras homologue expressed in brain), an activator of mTOR. The consequences of mTOR overexpression include abnormally rapid cell growth and hyperactivation of mRNA translation, which may lead to impaired synaptic plasticity in TSC [11] (Figure 1).

#### 2.1.3. Preclinical Models

Impaired synaptic plasticity as a consequence of a disruption in either TSC1 or TSC2 has been supported by results from preclinical studies. Abnormalities in long-term potentiation (LTP) and long-term depression (LTD) were found in the *Tsc2^+/^* ^−^ Eker rat, which carries a spontaneous germline mutation [12]. Abnormal late-phase LTP induction and hippocampal-dependent learning deficits was observed in *Tsc2^+/−^* adult mice and improved after rapamycin treatment [13]. 

Metabotropic glutamate receptor-mediated long-term depression (mGluR-LTD) was impaired in *Tsc2^+/^* ^−^ mice and related to decreased translation of proteins required for stabilization of LTD. Potentiation of mGluR5 activity led to restoration of normal LTD [14]. mGluR-LTD was also deficient in a neuronal model of *Tsc1* [15].

Prolonged neuronal hyperexcitability, typically associated with epilepsy, has also been supported by recent studies as a possible mechanism of impaired synaptic plasticity in TSC [16]. Hyperexcitability in cortical tubers has been linked to abnormalities of glutamate receptor expression [17]. This hyperexcitability was maintained despite the absence of cortical tubers from brain sections of an individual with TSC and *Tsc1^synapsin^ conditional*knockout mice, a neuronal model of TSC in an earlier study [18, 19]. An astrocyte-specific model of TSC, *Tsc1^GFAP^* conditional knockout mice, was also characterized by abnormally elevated glutamate [20]. Astrocytic dysfunction in the uptake of extracellular potassium may explain the hyperexcitability in this model [21].

#### 2.1.4. Clinical Trials

Guided by preclinical observations, investigators have completed studies to reduce the burden of neurologic disease in individuals with TSC. A clinical trial of everolimus for subependymal giant cell astrocytomas (SEGAs) achieved the primary outcome of reduction in the size of SEGAs, supporting similar results from a case series [22–24]. Everolimus is now FDA-approved for reduction in the size of SEGAs that are nonsurgically resectable. Positive outcomes from these studies have led investigators to consider rapamycin for additional neurologic conditions, such as autism [25]. The ability of everolimus to improve cognition is currently under investigation (http://www.clinicaltrials.gov/; NCT01289912).

### 2.2. Neurofibromatosis 1 (NF1)

#### 2.2.1. Clinical Features

Neurofibromatosis 1 (NF1), a disease caused by an inherited mutation in NF1, has an incidence of 1 in 3500 [26]. NF1 can be diagnosed by identification of the genetic mutation or the presence of two or more clinical features—family history of NF1; six or more cafe-au-lait spots; neurofibromas; plexiform neurofibromas; axillary or groin freckling; Lisch nodules (a hamartomatous nodule of melanocytes on the iris); skeletal abnormalities such as tibial dysplasia or thinning of the shin bone; or optic glioma. Associated conditions include cognitive impairments, pilocytic astrocytomas, and neuropathological abnormalities characterized by MRI hyperintensities, megalencephaly, and thalamic lesions. Cognitive impairment is the most common source of neurological impairment in children with NF1, affecting as many as 81% of children [27]. Neuropathological abnormalities associated with impaired cognition have been identified in some cases. Megalencephaly associated primarily with increased white matter volume was identified in individuals with NF1-associated ADHD [28]. Abnormalities in gray matter volume and enlargement of the corpus callosum have also been associated with cognitive impairment [29]. NF1 has also been characterized by the presence of MRI T2-hyperintensities (nonenhancing bright areas of unknown etiology), sometimes referred to as UBOs (unidentified bright objects). An early study employing sibling comparison found distribution of these lesions to be predictive of lower IQ [30]. Subsequent studies have also supported the role of these lesions in cognition [31, 32]. A longitudinal profile revealed changes in these lesions with childhood regression followed by recurrence in early adolescence [33].

#### 2.2.2. Neurobiology of mTOR Dysregulation

Disinhibited RAS MAPK signaling underlies the molecular basis of disease, and mTOR hyperactivity has also been identified in preclinical models [34]. NF1 encodes neurofibromin, a GTP-ase activating protein, which normally leads to inactivation of Ras. Mutations in neurofibromin lead to overactivation of Ras activity, followed by enhanced activation of the Ras-MAPK signaling pathway as well as PI3K and ERK 1/2 which both inactivate the TSC1/TSC2 complex releasing inhibition of Rheb and allowing activation of mTOR. However, there may be pathways leading to dysregulation of mTOR in NF1 that differ from other conditions [34]. mTOR hyperactivity in *Nf1 *leads to increased astrocyte proliferation, an effect not shared by preclinical models of *Pten*, *Tsc1*, *Tsc2*, or overexpression of *Rheb* [35]. Phospho-histone-H3 rather than phosphor-S6 or Ki67 correlated with response to rapamycin in *Nf1 *mice [36]. Neurofibromin also interacts with caveolin-1 [37] and nucleophosmin [38].

#### 2.2.3. Preclinical Models

Long-term potentiation was impaired by increased hippocampal inhibitory transmission in mice heterozygous for a germline mutation in *Nf1 *(*Nf1^+/−^*). However, restoration of LTP deficits and reversal of cognitive impairments was achieved with pharmacological inhibition of Ras using lovastatin, an HMG CoA reductase inhibitor [39] and BMS 191563, a farnesyltransferase inhibitor [40]. Farnesyltransferase inhibitors demonstrated inhibition of Rheb and subsequent inhibition of mTOR in *Tsc1^−/−^ and Tsc2^−/−^* mouse embryonic fibroblasts [41]. Inhibition of ERK also led to restoration of early-phase and long-term LTP [42].

#### 2.2.4. Clinical Trials

Simvastatin in children with NF1 improved object assembly, a secondary outcome in a randomized trial, but there was no difference in primary outcome [43]. Preliminary results of a subsequent of lovastatin in children with NF1 revealed improvement in verbal and nonverbal memory [44].

### 2.3. Fragile X Syndrome (FXS)

#### 2.3.1. Clinical Features

Fragile X syndrome (FXS) is the leading cause of inherited intellectual disability and has a full mutation gene frequency of 1 in 2500 [45, 46]. Associated neurologic conditions include autism, anxiety, and ADHD [47, 48]. Definitive diagnosis relies on genetic confirmation and individuals may be classified as full mutation if there are greater than 200 CGG repeats within the promoter of the fragile X mental retardation-1 gene (FMR1) and premutation if there are 50 to 230 repeats [49].

#### 2.3.2. Neurobiology of mTOR Dysregulation

These abnormal CGG repeats result in suppression of FMR1 gene transcription and subsequently reduced to absent fragile X mental retardation protein (FMRP) [50, 51]. Loss of FMRP releases inhibition of PIKE, which activates PI3K and leads to increased mTOR activity. The “mGluR theory” proposes that elevation of group I mGluRs (mGluR1 and mGluR5) glutamate receptors leading to reduced insertion of AMPA receptors into the postsynaptic membrane is one of the central mechanisms of impaired synaptic plasticity in FXS, and this has been supported in experimental models [52]. Increased mGluR5 activity and reduced insertion of AMPA receptors leads to long-term depression (LTD) due to reduced AMPA-mediated synaptic activity.

#### 2.3.3. Preclinical Models

Using preclinical models, specific interactions among synaptic proteins and FMRP have been identified. Initially, abnormal synaptic translation of CaMKIIa, PSD-95, and GluR1/2 mRNAs was observed in the Fmr1 knockout mouse [53]. Subsequent studies revealed regulation of expression of PSD-95 by FMRP, miR125a, and mGluR.

Phosphorylation of FMRP induces the creation of an AGO2-miR125a complex, which inhibits PSD-95 mRNA. mGluR stimulation, however, causes dephosphorylation of FMRP, which leads to activation of translation of PSD-95. In *Fmr1* KO mice, miR125a is reduced in addition to the reduction in FMRP [54]. In addition to hyperactivity of group 1 mGluR and mGluR-LTD, abnormally increased signaling of mTOR in hippocampus was discovered in *Fmr1 *KO mice, providing a link between mGluR elevation and abnormalities in synaptic plasticity leading to cognitive impairment. Loss of FMRP releases inhibition of PIKE, which activates PI3K and leads to increased mTOR activity as measured by four methods. Abnormally increased mTOR leads to an abnormal increase in cap-dependent translation of synaptic proteins and subsequent abnormalities in synaptic plasticity. Inhibition of PI3K activity resulted in normal levels of phosphorylated mTOR. Increased PTEN activity, mediated by dephosphorylation, was discovered in *Fmr1 *KO mice and may serve as a feedback inhibition to compensate for abnormally increased PI3K since PTEN dephosphorylates PI3K, which reduces phosphorylation and activation of Akt [55].

In a Drosophila model of FXS, treatment with mGluR antagonists during development resulted in reversal of neuropathology, abnormal courtship behavior, and impaired memory. Partial reversal of impaired memory and abnormal courtship behavior without change in neuropathology was seen in treated adults. Conversely, treatment led to impairment in wild-type control flies [56].

Reduction in genetic function of mGluR5, achieved by crossing *Fmr1 *mutant mice with heterozygous mGluR5 mutant mice, rescued many of the core phenotypic features in the *Fmr1* KO mouse [57]. Treatment of *Fmr1* KO mice with either an mGluR1 antagonist (JNJ) or an mGluR5 antagonist (MPEP) led to similar, but slightly different neurologic and behavioral improvements. Marble burying, a measure of repetitive behavior, was reduced without reduction in activity in *Fmr1* KO and WT mice. MPEP eliminated audiogenic-induced seizures. Motor learning also improved with MPEP in *Fmr1* KO mice. Prepulse inhibition, a measure of sensorimotor gating, known to be abnormally increased in *Fmr1* KO mice was not affected by JNJ or MPEP [58]. Abnormalities in prepulse inhibition were linked to abnormalities in presynaptic short-term plasticity in mice models of schizophrenia [59].

Prolonged UP states, a marker of cortical hyperexcitability in *Fmr1* KO mice was found to be due to a non-translation-related function of mGluR5, and treatment with MPEP reversed this phenomenon [60]. In addition to long-term postsynaptic plasticity, abnormalities in short-term presynaptic plasticity were also identified in *Fmr1* KO mice and may also contribute to cognitive impairment [61]. Another approach utilized GABA_A_ receptor agonist in *Fmr*1 KO mice, resulting in restoration of amygdala-based deficits in neuronal excitability, reduced prepulse inhibition, and alleviation of hyperactivity [62]. The behavioral effects of genetic reduction of mGluR1 and mGluR5 by 50% were observed in *Fmr1* KO mice. Reduction in mGluR1 led to decreased activity, whereas reduction in mGluR5 led to decreased active social behavior and decreased thermal sensitivity. Neither genetic reduction resulted in changes in memory, motor responses, sensorimotor gating, audiogenic seizures, and responses related to anxiety and perseveration [58].

#### 2.3.4. Clinical Trials

Human studies have led to the identification of the behavioral/cognitive profile of Fragile X as well an endophenotype of autism in Fragile X distinguished by social withdrawal [63–65]. Comparison of patients with FXS with and without autism supported the previously identified endophenotype of social withdrawal in FXS-associated autism by the finding of decrease in the left temporal gyrification index, an indicator of cortico-cortical connectivity and organization [66]. Recent significant scientific discoveries have culminated in human clinical trials targeting different aspects of the neurobiological impairments in FXS. AFQ056, an mGluR5 antagonist, resulted in different responses dependent upon the methylation status of FMR1. Patients with full methylation of FMR1 and no detectable FMR1 mRNA in the blood responded positively to treatment with improvement in inappropriate speech, stereotypic behavior, and hyperactivity [67]. Additional trials focused on antagonizing mGluR5 include a trial of fenobam, which reduced anxiety, hyperarousal, improved accuracy in continuous performance tasks, and prepulse inhibition of startle [68]; acamprosate, which in three young adult patients, resulted in improvement in communication and global clinical improvement (CGI-I) [69]. Results are pending from an open label phase I study of STX107 (Seaside Therapeutics) and a phase II trial of RO4917523 (Hoffman-LaRoche) (clinicaltrials.gov). Other mechanisms that may lead to repair of the impaired plasticity associated with FXS have also been examined. Phospholipase C and glycogen synthase kinase-3, linked to Gp1 mGluR signaling, have been targeted using lithium, which resulted in improvement in cognition and adaptive skills [70]. Ampalex (CX516) is an ampakine (binds AMPA receptors) that increases hippocampal LTP by slowing receptor deactivation [71, 72]. Evaluation of ampalex in a placebo-controlled phase II trial for Fragile X-associated autism did not reveal differences in the primary outcome of memory or any of the secondary outcomes: overall functioning, attention/executive functioning, language, or behavior [73]. Minocycline, a broad spectrum antibiotic and analogue of tetracycline, has been found to have neuronal effects. In C57BL/6J mice, minocycline increased phosphorylation of GluR1 and subsequent insertion of AMPA receptors *in vivo* and *vitro* [74]. Study of minocycline in *Fmr1* KO mice revealed behavioral improvement: reduction in anxiety and improved exploration as well as neuropathological improvement—dendritic spine maturation associated with inhibition of abnormally elevated matrix metalloproteinase-9 (MMP-9) in hippocampal neurons [75]. The observations in *Fmr1* KO mice were supported in a Drosophila model of FXS where treatment with minocycline or genetic elimination of mmp1 reverses synaptic structural abnormalities [76]. Open-label treatment with minocycline in individuals with FXS led to significant improvement in irritability [77]. Cholinergic deficits in FXS, confirmed in individuals by ^1^H magnetic resonance spectroscopy, were targeted using donepezil in an open label study with noted improvement in continuous naming, attention difficulties, and total ABC score as well as irritability and hyperactivity [78]. Reduction in glutamate using riluzole in an open-label study corrected abnormal activation of ERK; however, improvement in the primary outcome-repetitive, compulsive behavior was not achieved [79]. A single-dose, placebo-controlled trial of oxytocin for social anxiety in FXS resulted in improvement in eye gaze towards the examiner in a social challenge [80]. Aripiprazole, an atypical antipsychotic that is a partial D2 and 5-HT1A agonist as well as a 5-HT2A antagonist, improved scores on CGI-I and ABC-irritability [81]. Baclofen, a GABA_B_ receptor agonist, inhibited seizures in *Fmr1* mice [82]. A phase II, randomized double-blind study of arbaclofen has been completed with results pending, and a phase III study of arbaclofen is now recruiting (http://www.clinicaltrials.gov/).

### 2.4. PTEN-Associated Conditions

#### 2.4.1. Clinical Features

Phosphatase and tensin homologue deleted on chromosome 10 (PTEN) is a phosphatase which limits cell growth by apoptosis and cell cycle arrest. Conditions linked by a genetic mutation in PTEN have been collectively termed phosphatase and tensin homologue hamartoma syndromes (PTHS) and include Juvenile Polyposis, Lhermitte-Duclos disease, Bannayan-Riley-Ruvalcaba, Cowden Syndrome, Proteus-syndrome, and Proteus-like conditions. Cowden syndrome and Bannayan-Riley-Ruvalcaba have been associated with autism and intellectual disability.

Cowden syndrome has an estimated prevalence of 1/200,000 and may be diagnosed by the presence of either pathognomonic criteria or a specific combination of major and minor criteria. Severe and progressive macrocephaly (>2 S.D.) associated with autism should prompt consideration of the diagnosis and led to publication of the first reported case of Cowden syndrome-associated autism and epilepsy [83]. A similar pattern with the addition of a lipoma and thyroid adenoma led to the identification of Bannayan-Riley-Ruvalcaba Syndrome (BRRS) in a nine-year-old girl [84]. A retrospective review of 114 patients analyzed for PTEN mutations revealed mutations in 18% of those with macrocephaly in addition to either ASD or ID [85]. This contrasts with a PTEN mutation in one of eighty-eight children (1%) with macrocephaly and ASD [86]. BRRS does not have established diagnostic criteria; however, macrocephaly is usually the most striking feature. Identification of Cowden and Bannayan-Riley-Ruvalcaba syndrome in the same family raises the possibility of the two syndromes being the same syndrome with variation in phenotypic expression [87].

Functional analysis of the consequence of PTEN germline mutations from individuals with autism spectrum disorders was compared to PTEN germline mutations in individuals with PTHS in a humanized yeast-based bioassay and revealed greater preservation of PTEN PIP3 phosphatase activity in those with ASD [88].

#### 2.4.2. Neurobiology of mTOR Dysregulation

PTEN is important in mTOR signaling since it removes a phosphate from phosphatidylinositol 3,4,5-triphosphate (PIP3). This conversion from PIP3 to PIP2 negates the activity of PI3K and results in elevation of mTOR since the processes downstream—Akt activation, Akt-mediated phosphorylation and inhibition of TSC2, release inhibition of Rheb which activates mTOR.

#### 2.4.3. Preclinical Models

Evidence of impaired synaptic plasticity in PTEN mutations has been identified in *Pten* conditional knockout mice. Neuropathological features include enlarged neuronal nuclei and cell bodies, increased density of dendritic spines, abnormalities in axonal myelination, and weakening of excitatory synaptic transmission in hippocampal neurons between CA3 and CA1 as evidenced by impaired EPSPs, normal presynaptic function, and reduced long-term potentiation [89]. Cre-driven deletion of *Pten* in cortical and hippocampal neurons of mice was associated with hyperactivity of the mTOR pathway as well as hypersensitivity to stimuli, social interaction abnormalities, ectopic dendrites, increased axonal synapses, and macrocephaly associated with neuronal hypertrophy [90].

#### 2.4.4. Clinical Trials

A pilot study is now recruiting for an open label trial of sirolimus, an mTOR inhibitor, in adult patients with Cowden syndrome, tumors, and germline PTEN mutations (http://www.clinicaltrials.gov/).

### 2.5. EIF4E-Associated Autism

Synaptic translation mediated by EIF4E is a common and final process of the pathways associated with PTEN, mTOR, and FMRP and serves a critical role in learning and memory [91, 92]. Linkage to chromosome 4q, the region containing EIF4E has been shown in genome-wide linkage studies [93, 94]. After identification of a translocation involving the region containing EIF4E in a young boy with autistic regression, investigators screened for mutations among families with two autistic siblings and found EIF4E mutations in two related families [95].

## 3. Conclusions

Review of recent literature reveals significant advances in our ability to understand the pathogenesis of several neurogenetic conditions associated with intellectual disability and autism that have been considered to be idiopathic and untreatable. In this paper, we have highlighted recent discoveries in neurogenetic conditions united primarily by dysregulation of mTOR and evidence of impaired synaptic plasticity (Table 2). In addition to autism and intellectual disability, some of these conditions also share an association with cutaneous lesions and tumor development. Based on this knowledge, it is reasonable to hope that these disorders could become treatable in the near future. Investigators have already begun the process of connected research, as exemplified by the work of Auerbach et al. who simultaneously examined models of TSC and FXS and created a model by crossing the two models to discover that the same intervention, modulating metabotropic glutamate receptor 5, demonstrates efficacy for both models in opposing directions [14]. Continuing to examine the link between these disorders is likely to lead to a greater chance of discovery for all of them. Tools needed to translate basic science research into clinical trials which yield definitive results include refined genotypic and phenotypic characterization, detailed knowledge of the natural history of the conditions, knowledge of optimal therapeutic windows, valid biomarkers, and expertise in clinical trials. 

## Figures and Tables

**Table:** (a) TSC. Definite TSC: two major or one major and two minor features; probable; TSC: one major and one minor feature; possible TSC: one major or two or more minor features

Major features	Minor features
Cortical tubers	Dental enamel pits
Subependymal nodules	Hamartomatous rectal polyps
Subependymal giant cell astrocytoma	Bone cysts
Hypomelanotic macules (3 or more)	Cerebral white matter radial migration lines
Shagreen patch	Gingival fibromas
Facial angiofibromas or forehead plaque	Nonrenal hamartoma
Multiple renal nodular hamartomas	Retinal achromatic patches
Nontraumatic ungual or periungual fibromas	“Confetti” skin lesions
Cardiac rhabdomyoma	Multiple renal cysts
Pulmonary lymphangiomyomatosis and/or renal angiomyolipomas	

**Table:** (b) NF1. Presence of two or more clinical features

Family history of NF1	Neurofibromas or plexiform neurofibromas
Six or more cafe-au-lait spots	Axillary or groin freckling
Lisch nodules	Skeletal abnormalities—tibial dysplasia or shin bone thinning
Optic glioma	

**Table:** (c) FXS

Full mutation >200 CGG repeats	
Premutation 50–230 CGG repeats	

**Table:** (d) PTHS (Only Cowden syndrome has diagnostic criteria). Cowden syndrome. Operational diagnosis: mucocutaneous lesion alone if: 6 or more facial papules, 3 or more trichilemmoma; cutaneous facial papules and oral mucosal papillomatosis; oral mucosal papillomatosis and acral keratosis, or 6 or more palmoplantar keratosis; or two or major criteria, including macrocephaly or adult Lhermitte-Duclos disease; or one major or three minor criteria; or four minor criteria

Pathognomic criteria	Major criteria	Minor criteria
Adult Lhermitte-Duclos	Breast cancer	Intellectual disability
Mucocutaneous lesions	Thyroid cancer	Other thyroid lesions
	Macrocephaly	GI hamartomas
	Endometrial cancer	Fibrocystic breast disease
		Lipomas; fibromas
		Genitourinary tumors or malformations

EIF4E (No diagnostic criteria).

**Table 2:** Mechanisms of impaired synaptic plasticity, mTOR dysregulation, and therapeutic targets.

Condition	Gene (chromosome)	Mechanism of impaired synaptic plasticity impairment	mTOR physiology	Therapeutic targets
TSC	TSC 1 (9) or TSC2 (16)	↓mGluR-LTD	↑mTOR	mTOR antagonists mGluR 5 agonist

NF1	NF1 (17)	↓LTP↑GABA	↑mTOR	Ras antagonists ERK antagonists

FXS	FMR1 (X)	↑mGluR-LTD	↑mTOR	mGluR5 antagonists mGluR5/mGluR1 genetic reduction GABA_A_ receptor agonist PLC/GSK3 antagonist (lithium) AMPA receptor agonist MMP 9 antagonist

PTHS	PTEN (10)	↓LTP	↑mTOR	mTOR antagonists

EIF4E-associated autism	EIF4E (4)	unknown	Downstream of mTOR	None developed
